# A Comprehensive Overview of *Candida albicans* as the Leading Pathogen in Vulvovaginal Candidiasis

**DOI:** 10.3390/jof11090632

**Published:** 2025-08-28

**Authors:** Nika Srb, Jasminka Talapko, Tomislav Meštrović, Rajko Fureš, Mirjana Stupnišek, Andrea Milostić Srb, Ivana Škrlec

**Affiliations:** 1 Faculty of Dental Medicine and Health, Josip Juraj Strossmayer University of Osijek, 31000 Osijek, Croatiajtalapko@fdmz.hr (J.T.); rajko.fures@gmail.com (R.F.); mstupnisek@fdmz.hr (M.S.); 2 University Centre Varaždin, University North, 42000 Varaždin, Croatia; tmestrovic@unin.hr; 3 Institute for Health Metrics and Evaluation, University of Washington, 3980 15th Ave. NE, Seattle, WA 98195, USA; 4 Department of Gynecology and Obstetrics, Zabok General Hospital and Croatian Veterans Hospital, 49210 Zabok, Croatia; 5 Faculty of Medicine, Josip Juraj Strossmayer University of Osijek, 31000 Osijek, Croatia

**Keywords:** *Candida*, *Candida albicans*, vulvovaginal candidiasis, VVC, vaginal fungal infections

## Abstract

*Candida albicans* is the primary etiological agent of vulvovaginal candidiasis (VVC), a widespread fungal infection affecting millions of women worldwide. Although often self-limiting, VVC can become recurrent or severe, significantly impacting quality of life. The pathogenesis of *C. albicans* is driven by key virulence factors, including hyphal transformation, biofilm formation, and immune evasion, which all facilitate persistence and resistance to host defenses. Epidemiological data indicate that up to 75% of women experience at least one episode of VVC, with 5–10% developing recurrent vulvovaginal candidiasis. The condition typically presents with vaginal itching, burning, erythema, edema, and an abnormal discharge. Diagnosis relies on both clinical presentation and microbiological confirmation; however, misdiagnosis remains common due to symptom overlap with other vaginal infections and conditions in general. Azole antifungals remain the cornerstone of treatment; however, increasing resistance (particularly in non-albicans *Candida* species) poses substantial therapeutic challenges. Consequently, the emergence of antifungal-resistant strains underscores the need for novel treatment strategies, including probiotics and natural antifungal agents. Preventive measures—including maintaining vaginal microbiota balance, avoiding unnecessary antibiotic usage, and improving hygiene practices—play a pivotal role in reducing disease burden due to *C. albicans*. Given the rising incidence of VVC and the burden of recurrent cases, further research is essential to develop targeted therapeutic interventions. This comprehensive review highlights the evolving epidemiology, pathogenesis, and clinical challenges of *C. albicans*-associated VVC, emphasizing the need for improved diagnostic strategies, alternative therapeutic approaches, and targeted preventive measures to reduce disease burden and enhance patient outcomes.

## 1. Introduction

In the first half of the 20th century, fungal infections were diagnosed less frequently and deemed of lower clinical significance than viral and bacterial infections [1,2]. However, with the rise in immunocompromised patients due to infections with human immunodeficiency virus (HIV) or in the context of malignant diseases, the awareness of the dangers of fungal infections has risen [1,3]. The two types of mycotic infections are (1) superficial infections (skin and mucous membranes) and (2) invasive fungal infections [3]. The prevalence of fungal infections is on the rise all over the world; more specifically, around 300 million people are infected every year, and over 1.7 million die as a result of these infections [1,3]. The most common cause of mycotic infections are species of the genus *Candida*, which are also one of the leading causes of healthcare-associated infections (HCAI) [3,4]. The *Candida* genus includes a variety of species, and the ones responsible for over 90% of all *Candida* related diseases are *Candida albicans*, *Candidozyma auris* (*Candida auris*), *Pichia kudriavzevii* (*Candida krusei*), *Nakaseomyces glebaratus* (*Candida glabrata*), *Candida parapsilosis*, and *Candida tropicalis* (Table 1) [3,5].

## 2. *Candida albicans*

**Figure 1:**
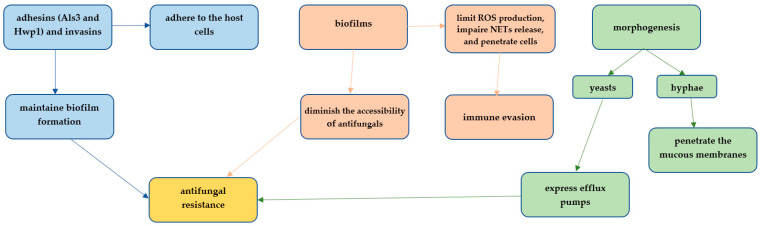
Key virulence traits of *Candida albicans*. NETs—Neutrophil Extracellular Traps; ROS—Reactive Oxygen Species. Information was collected from the following references [20,34,35,36,37,38,39,40].

The paradigmatic name *Candida albicans* refers in both parts to the color white and the fungi itself, which is an opportunistic human commensal [7,8]. Its genome is around 16 MB in size and has eight sets of chromosomes (chr1A-chr7A and chrRA) when haploid and 28 MB when diploid (chr1B, chr2B…, chrRB) [9,10]. Around 97% of its CTG codons translate as serine and 3% as leucine with an elevated tolerance for the misincorporation of leucine at CTG codons, which is caused by stress and affects the functioning of key signaling molecules of pathogenesis dramatically; it also masks β-glucan in the cell wall and interferes with host recognition [5]. Gene families responsible for *C. albicans* virulence are oligopeptides and iron transfer proteins, aspartyl proteases and lipases, and the ALS (agglutinin-like sequence) adhesins [5]. Adherence is possible due to SAP1 and SAP4 (secreted aspartic proteinase) promoting the invasion of host tissues by breaking down cell surface proteins [11]. The main transcription regulator of biofilm formation is EFG1 (enhanced filamentous growth protein), while ZAP1 (zinc-responsive transcription factor) is involved in biofilm maturation [12]. Additionally, ALS3 (Agglutinin-like protein 3) enables adherence to plastic (i.e., biofilm development) and binds to the host’s N-cadherin and E-cadherin, leading to endocytosis of *C. albicans* (Figure 1). Furthermore, when it binds to the EGF (epidermal growth factor) receptor and HER2 in epithelial cells, it induces autophosphorylation of those receptors and endocytosis of the fungi [5]. These proteins—and others such as Hwp1 (Hyphal wall protein 1), Als9 (Agglutinin-Like Sequence 9), and Als1-7 (Agglutinin-Like Sequence 1-7), together with β-glucan and chitin—make up the cell wall [13,14]. *C. albicans* wall is dynamic, which allows it to adapt to the environment. Still, the wall also protects the organism from environmental changes, such as stress, changes in osmosis, temperature, and immune attacks by the host or other organisms [15]. The walls are stable thanks to the sterols in the cell membrane, the most common being ergosterol, which is synthesized in the endoplasmic reticulum (ER) [16,17]. Glucose serves as a carbon source, and amino acids provide a source of nitrogen to meet the nutritional needs of the fungus [18]. Another virulence factor is Candidalysin, a cytolytic peptide toxin that directly damages epithelial membranes, triggers a danger response signaling pathway and activates epithelial immunity [19].

Morphologically, *C. albicans* can be in the form of asexual blastopores that divide by budding, as hyphae that are branched chains of tubular cells, or as chains of elongated yeast cells called pseudohyphae [20,21]. The yeast form is the type present in the human microbiome [22]. The switch from the yeast form to the invasive hyphae or pseudohyphae is associated with virulence, as hyphae are able to invade and damage epithelial and endothelial cells, allowing the fungus to evade host macrophages and escape phagocytosis [5,23,24]. The transition from round white cells to elongated opaque cells contributes to virulence and is associated with mating [25]. The white cells lead more to systemic infections, while opaque cells cause more skin infections and seem better at evading the host immune response [5]. This transition ability enables a quick response to environmental changes by leading to differences in filamentous growth and interactions with immunological cells. It is also transcriptionally stable, even for many generations [26,27].

The fungus has the ability to produce a biofilm, which consists of various morphological cell types (round yeast cells, elongated/cylindrical hyphal cells, and oval pseudohyphal cells) due to its ability to transition from yeast to hyphae [28,29]. Biofilm development occurs in three phases, beginning with the adherence of *C. albicans* to the substrate surface, where it forms the basal layer. In the next phase, elongated protrusions are formed by the cells that grow into filamentous hyphal forms [30]. Then, the extracellular polysaccharide matrix accumulation ensues in the maturation phase. At the same time, the last phase involves the dispersion of non-adherent cells, which may disseminate and form novel biofilms [14]. Biofilm is worrisome because it can adhere to various surfaces in medical settings—from contact lenses, dentures, joint prostheses, and urinary and central venous catheters to pacemakers and mechanical heart valves. Moreover, it can also be disseminated into the bloodstream, leading to invasive systemic infections [28]. Such infections occur in around 5% of all patients with catheters. They may lead to deadly outcomes since high rates of resistance to antifungals are observed (which is compounded in biofilms). At the same time, removing the infected device, like heat valves, is dangerous and complicated [28,31]. Changes also happen in the expression of genes that are indirectly involved with biofilm formation. The cells in the deeper layers of the biofilm increase their expression of genes related to the metabolism of sulfur-containing amino acids, which enables them to survive starvation and oxidative stress since sulfur amino acids are involved in synthesis of antioxidants [32]. An increase in the expression of genes related to glycolysis, fatty acid metabolism, and ergosterol synthesis also occurs [33].

### Diseases Caused by Candida albicans

*C. albicans* is part of the local microbiota in healthy individuals. As such, it poses no threat unless the immune system is weakened, leading to fungal overgrowth and, potentially, the initiation of an infectious process [28,41]. Immunodeficiency can be caused by bacterial or viral infections, stress, and various other diseases (organ malignancies, burns), and even treatments like antimicrobial agents, hemodialysis, immunosuppressive agents, or major surgeries [28,42]. Other medical risk factors include a prolonged hospital stay and admission to the intensive care unit (ICU) [42,43]. Infections can range from mild, superficial mucosal and dermal infections, such as thrush, diaper rash, and vaginal yeast infections, to highly lethal, blood-disseminated infections [28]. A special risk group of immunocompromised patients includes those suffering from acquired immunodeficiency syndrome (AIDS) or undergoing anticancer treatments [28]. The invasive and dangerous diseases caused by *C. albicans* include candidemia, CNS candidiasis, urinary candidiasis, endophthalmitis and chorioretinitis, *Candida* endocarditis, bone and joint candidiasis (spondylodiscitis, osteomyelitis, arthritis, and prosthetic joint infections), and *Candida* pneumonia [39,41].

## 3. Vulvovaginal Candidiasis

Vulvovaginitis is defined as an inflammation of the vulva and vagina, commonly caused by bacteria, *Trichomonas vaginalis*, yeast infections, or by non-infectious causes such as allergic reactions and atrophic vaginitis that is frequently observed in menopause [44,45]. Of the mentioned causes, opportunistic fungal infections are usually the most common, specifically caused by *Candida albicans*, but may also be caused by other *Candida* species (Table 1) or different yeasts [44,46]. In cases of *Candida* infection, we are referring to vulvovaginal candidiasis (VVC), which accounts for approximately one-third of all vulvovaginitis cases [44,47]. Since *C. albicans* is a commensal species, certain conditions must occur for it to transition to a pathogenic state, such as vaginal dysbiosis, evasion of the immune system, and high expression of virulence factors (hyphal growth, biofilm formation, production of proteolytic enzymes, and candidalysin) [48].

### 3.1. Pathophysiology

**Figure 2:**
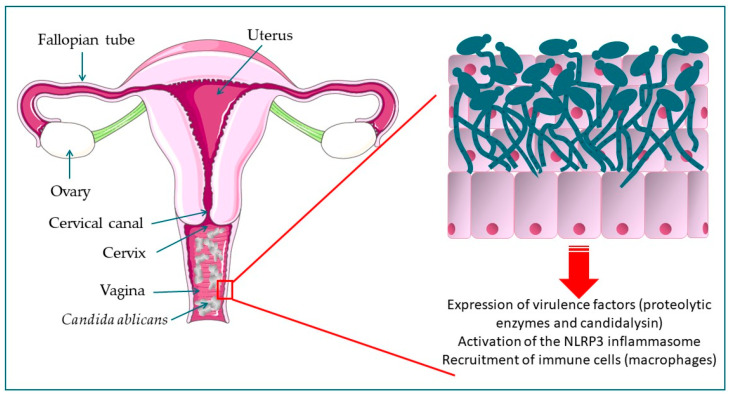
Pathophysiological process in the development of vulvovaginal candidiasis. The onset of VVC is triggered by the secretion of cytotoxic proteins of *C. albicans* (proteolytic enzymes and candidalysin) and the activation of the NLRP3 inflammasome signaling pathway, which leads to an inflammatory response (production of cytokines and chemokines by epithelial cells). The recruitment of cells belonging to the innate immune system (polymorphonuclear cells and macrophages) within the vaginal canal is the primary cause of the clinical symptoms associated with *C. albicans* infection. The transition from yeast to hyphae triggers NLRP3 activation, rendering the NLRP3 inflammasome a crucial contributor to host immunopathology.

As part of the typical human microbiota, *C. albicans* is a common vaginal colonizer that is present in the lumen and usually causes no symptoms [49]. Vulvovaginal candidiasis stars when *Candida* spp. pierces the mucosal linings of the vagina, leading to an inflammatory response induced by its pseudohyphae and hyphae that activate the NLRP3 (NOD-like receptor protein 3) inflammasome in epithelial cells [44,48]. Polymorphonuclear cells and macrophages are usually the primary inflammatory cells, and the inflammatory response causes a dense discharge, irritation, itching, burning, excoriations, dysuria, dyspareunia, and even swelling [44] (Figure 2). Although it is known that *C. albicans* causes neutrophil infiltration, the underlying mechanism remains undetermined. A recent study found that levels of proinflammatory cytokines (IL-1β and IL-8) are favorably connected with the expression of the PRA1 (pH-regulated antigen) gene, which is significantly elevated during vaginal candidiasis [50]. The gene is responsible for encoding a protein with various immune-modulatory properties, such as neutrophil chemotaxis and complement modulation [50].

Hyperglycemia enables *Candida* establishment and adhesion to proliferate in tissues, vessels, and mucosa, increasing the fungi’s growth [51]. This, in turn, compromises neutrophil migration and leads to impaired chemotaxis and phagocytosis [51]. Studies also suggest that high glucose levels may enhance the expression of C3 fungal receptors and increase biofilm production in various fungi, including *Candida albicans* [51,52,53,54].

The main virulence factor of *C. albicans* is the ability to switch between its two major morphological forms—the ovoid yeast and the elongated hypha. Without this ability, this fungal species could rarely lead to pathogenicity or even colonization [13,49,55]. Studies of vulvovaginal candidiasis conducted on mice revealed that hypha-deficient transcriptional regulator mutants showed higher colonization than hypha-competent strains [49].

### 3.2. Epidemiology

When it comes to vulvovaginitis, data suggests that 75% of women experience it due to *Candida* spp., 40–45% have two or more episodes, and 5–10% of all women will develop recurrent vulvovaginal candidiasis (RVVC) [46,48,56]. VVC can be found in 10–15% of asymptomatic women, as it is a disease primarily affecting immunocompetent and healthy women, unlike systemic and oral candidiasis [56,57]. A systematic review by Denning et al. concluded that, on a global scale, VVC affects approximately 138 million women each year (range: 103–172 million), with a prevalence rate of 3871 per 100,000 women [58,59]. They also suggested that the age group of 25–34 has the highest overall prevalence (9%) and that 372 million women suffer from RVVC during their lifetime [58]. Risk factors can be divided into host-related and behavioral risk factors [56]. Host-related factors include pregnant women and women using hormone replacement therapy (HRT), diabetes mellitus, immunosuppression, antibiotic overuse, use of glucocorticoids, and genetic predispositions [56,58]. Antibiotics can perturb the composition of the normal vaginal microbiota, leading to *C. albicans* overgrowth in the gastrointestinal tract, vagina, or both [60]. The higher incidence of vulvovaginal candidiasis during gestation is due to the higher secretion of sex hormones in pregnancy, especially in the last trimester. At the same time, recurrences are commonly observed throughout pregnancy [56]. Similarly, in nonpregnant women, the incidence is higher during the luteal phase of the menstrual cycle, which is the phase of the highest hormone secretion [56]. Behavioral risk factors encompass the usage of oral contraceptives or intrauterine devices, hygiene habits, consumption of sugar-rich food, contact with allergenic chemicals such as douches, sexual practices, and new sexual partners. However, risk factors can vary; sometimes, they may be idiopathic [56,58,60].

### 3.3. Clinical Manifestations

The problem with VVC is that none of the frequently occurring clinical symptoms of VVC are specific and are often associated with a variety of other vaginal diseases, especially sexually transmitted ones like bacterial vaginosis, gonorrhea, and trichomoniasis [46,56]. The most pervasive symptoms and signs are vaginal soreness, genital discomfort (including pruritus), a burning sensation followed by vaginal soreness and irritation that leads to dyspareunia and dysuria, often accompanied by an abnormal vaginal discharge [46,48,56]. Other common symptoms/signs are erythema of the vulva and vagina, edema, and fissures [56].

VVC can be classified as uncomplicated or complicated based on clinical presentation, host factors, microbiology, as well as response to therapy [46] (Table 2). Uncomplicated VVC are sporadic or infrequent VVC, mild-to-moderate VVC, and is likely to be caused by *C. albicans* [46]. Complicated VVC affects around 10–20% of women and can be divided into recurrent VVC, severe VVC, and non-albicans VVC, which all require special diagnostic and therapeutic considerations [46,60]. Recurrent vulvovaginal candidiasis is defined as four or more episodes of symptomatic VVC in one year and has an incidence of 5–10% [48,60]. Recurrent VVC symptoms vary in severity from moderate to severe, but all types affect life quality and are associated with high amounts of stress [58]. A study conducted in 2020 found that VVC was related to higher levels of anxiety and depression among those suffering from recurrent forms of the disease [61].

Along with the typical symptoms of itching, soreness, and discomfort, recurrent VVC also leads to loss of confidence and self-esteem, limits daily physical activities, and negatively impacts sexual life and intimate relationships [58]. Due to recurrent VVC, male partners may suffer from penile irritation, and there are often suspicions about sexually transmitted diseases acquired from their partners [58]. The other type of complicated VVC, severe vulvovaginal candidiasis, occurs when the patients have poorer responses to therapy and include symptoms such as extensive vulvar erythema, prominent edema, excoriation, and fissure formations [60].

### 3.4. Diagnostic Considerations

**Figure 3:**
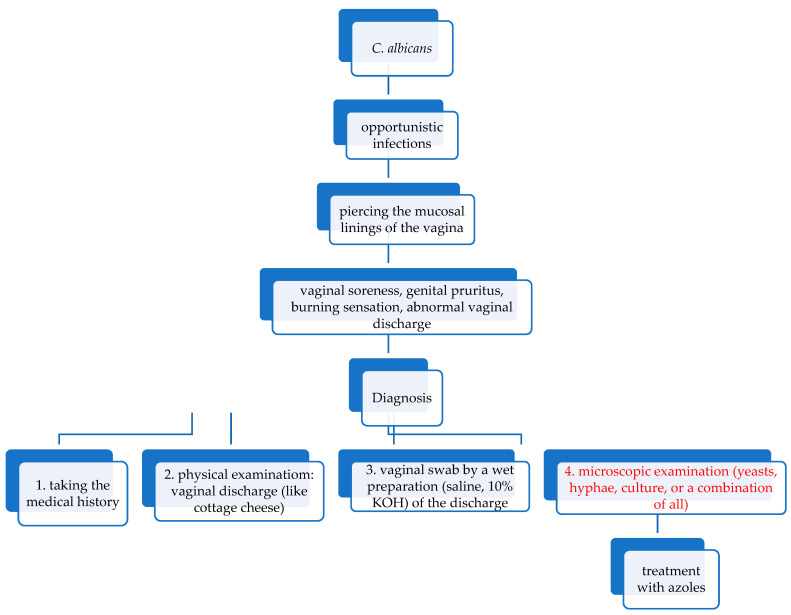
A flowchart demonstrating the pathway of the infection.

When VVC is concerned, the diagnosis is clinically indicated by the sudden appearance of vulvar pruritus and pain, external dysuria, edema, and erythema [46]. However, the majority of healthy women, especially with uncomplicated vulvovaginal candidiasis, are asymptomatic [46]. Vulvovaginal candidiasis is diagnosed initially by taking a medical history, where patients mention pruritus as the most typical symptom [60]. The physical examination reveals vaginal discharge, which is homogenous and serous-mucous in consistency, usually minimal, described as looking like cottage cheese, and having minimal odor [60]. Suppose these clinical signs and symptoms are present. In that case, VVC diagnosis is confirmed by a combination of microscopic examination showing yeasts, hyphae, culture, or a combination of all from a vulval or vaginal swab by performing a wet preparation (saline, 10% KOH) of the vaginal discharge [46,60,62]. Potassium hydroxide (10% KOH) disrupts cellular substances that may conceal the yeast or pseudohyphae, thus improving the visualization of yeast and mycelia [46]. Every patient who exhibits signs and symptoms of fungal vaginitis should undergo examination with a wet mount and KOH preparation (Figure 3). A study by Zhao et al. suggested that FB 85 (fluorescent brightener 85) could be used as a fluorescent substance for diagnosing VVC instead of KOH [63]. The patients should undergo treatment if they return with a positive result [46]. If, for some reason, *Candida* cultures cannot be prepared, empiric treatment can be applied [46].

Recently, matrix-assisted laser desorption ionization mass spectrometry (MALDI-TOF MS) has been used as an alternative method for the biochemical and molecular identification of *Candida* species [64]. Namely, it is a reliable method used in clinical laboratories for routinely identifying fungi and other microorganisms, (especially bacteria) with characteristic mass spectra of ribosomal proteins and compare them to reference mass spectra in the database. This method, from intact microorganisms, facilitates the identification of a wide range of proteins [64].

### 3.5. Treatment

The effectiveness of treatment is influenced by both the time of diagnosis and the time of treatment initiation [42].

The treatment of VVC usually relies on topical and/or oral antifungals, with azoles being the most commonly used treatment modality [48]. Studies show that completed azole treatment relieves symptoms and reduces negative cultures in 80–90% of cases [46]. Systemic azoles and triazoles include itraconazole, fluconazole, voriconazole, posaconazole, and isavuconazole [42]. The choice of which drug to use often depends on individual factors like the severity of the infection and whether it is a recurring issue. However, topically applied clotrimazole is the most frequently prescribed for uncomplicated infections. The azoles mechanism of action works by inhibiting the key enzyme in ergosterol biosynthesis, lanosterol 14α-demethylase, thus interfering with fungal cell wall synthesis [42,48]. However, of all the mentioned azoles, the drug of choice for fungal urinary tract infections is fluconazole since it achieves high concentrations in urine, unlike the other azoles [65,66]. Their effectiveness against *Candida* spp. stems from their high fungistatic activity [42]. Another advantage of azoles is that they are generally well-tolerated. Aside from VVC, azoles are first-line therapy for other forms of deep-seated candidiasis, including urinary tract infections and intraocular and central nervous system infections, since they easily cross the blood–brain barrier [42,67]. Azoles can be administered orally, intravenously, or locally [46,67]. Uncomplicated vulvovaginal candidiasis is successfully treated with short-course topical azoles, used in a single dose or continuously for 7–14 days [46]. The most common treatment modalities and their corresponding treatment durations are listed in Table 3. VVC is not a sexually transmitted disease; therefore, male partners do not require medical treatment [46]. Some men, however, may suffer from balanitis, characterized by redness of the glans penis along with pruritus and/or irritation; therefore, the use of topical antifungals may relieve their symptoms [46].

Treatment of VVC can be challenging due to the inherent resilient nature of fungi, which contributes to the recurrence of infections [48]. Complicated VVC (such as recurrent VVC) responds well to short-duration therapy with oral or topical azoles [46]. Still, to maintain clinical and mycologic control, a longer duration of therapy is necessary to achieve mycologic remission [46]. Typically, fluconazole is prescribed orally to be taken once a week for 6 months. In cases of treatment failure, topical treatment should be applied intermittently, and experts also recommend a twice-weekly dosing schedule and performing culture and drug susceptibility test as well [46,68]. Suppressive therapy is also used to control recurrent VVC and usually provides complete withdrawal of symptoms for the duration of the treatment [46,58]. When suppressive maintenance is completed, infections may still occasionally occur, and some patients may even require additional rounds of suppressive therapy, often for prolonged periods [58]. According to data, women who have significant vaginal excoriation, prolonged symptoms duration, and atopic disease are less likely to respond to the fluconazole maintenance treatment [69]. Aside from fluconazole, itraconazole can also be prescribed [58]. From all this, it can be deduced that suppressive therapy is rarely curative long-term [46]. Another complicated VVC is severe vulvovaginal candidiasis, characterized by widespread vulvar erythema, edema, excoriations, and fissures [46]. Treatment of severe VVC has lower clinical success rates when administered orally or topically for a short period [46]. Thus, the recommended period is topical use of azoles for 1–2 weeks or oral administration of 150 mg of fluconazole in two sequential doses [46]. Although they may not seem significant, there are structural variations between azoles that determine their bioavailability, interactions, antifungal spectrum, potency, and toxicity [37]. According to a 2018 meta-analysis, the most effective antifungal agents for treating VVC were fluconazole, followed by butoconazole, terconazole, clotrimazole, itraconazole, econazole, and miconazole [70].

Even though there are many benefits to using azoles, as with every drug, there can also be side effects. Chronic use may lead to hepatotoxicity (abnormal elevations of liver enzymes), as well as hormone-related adverse effects such as alopecia, decreased libido, gynecomastia, oligospermia, azoospermia, impotence, hypokalemia, hyponatremia, and, although rare, even adrenal insufficiency may occur [46,71,72]. Orally administered azoles sometimes lead to nausea, abdominal pain, and headaches [46]. Most of these serious adverse effects are associated with systemic use. The potential of oral azoles to cause teratogenicity is another limitation. For this reason, oteseconazole is only recommended for women who are not of childbearing age and topical azoles are advised for pregnant women [73,74]. Thus, azoles are generally safe when used topically to treat VVC; however, local burning or irritation may occasionally occur [46]. Due to the structural similarities between cholesterol and ergosterol, azoles may also have off-target effects on human cells, which could result in adverse effects such as liver damage or drug interactions [75]. There are also many clinically significant interactions when oral azoles are used in combination with other drugs [46]. For example, azoles lower/inhibit the metabolism of HMG-CoA reductase inhibitors, cisapride, cyclosporine, phenytoin, and carbamazepine [76]. Drugs such as barbiturates and rifampicin increase the metabolism of azoles, whereas H2-receptor antagonists decrease their metabolism [76].

Furthermore, the extensive and repeated use of antifungals as prophylaxis and therapy has led to the rise in resistant *Candida* spp. [48]. The mechanisms of resistance include the upregulation or mutation of genes, such as ERG11, which encodes ergosterol, a target of antifungal drugs. Additionally, genes like CDR1/2 and MDR1 are involved in expelling medications through the multidrug efflux pump in fungal cell walls, or genes that modify cell walls and membranes [77]. Research shows that efflux pumps are the most common and dominant mechanism of azole resistance in all *Candida* spp. [78,79]. Risk factors for the emergence of resistance include advanced age, a compromised immune system, prior medical conditions, and severe immunosuppression (such as following organ transplantation) [80]. The emerging resistance is mostly to fluconazole. Although resistance to other azoles is rare, it also increases in frequency [42]. Species such as *C. auris*, and *C. krusei*, have intrinsic resistance to fluconazole [42]. This intrinsic fluconazole resistance is another problem that occurs since VVC caused by *C. glabrata* is also on the rise [42,58]. Fluconazole is still the first-line treatment option in developing countries with a low prevalence of azole resistance for all invasive *Candida* infections [42]. When it comes to treating women with azole-resistant VVC, the use of itraconazole orally is recommended [81]. However, if cross-resistance occurs to either of these two, there is no other approved oral azole currently available that is effective against *C. albicans* [81]. When cross-resistance to oral agents occurs, resistance to all azole drugs is likely to occur [81]. As the resistance of *C. albicans* to azoles increases, susceptibility tests, if available, should be obtained for symptomatic women who continue to test positive for *C. albicans* despite maintenance therapy [46]. Studies show that voriconazole can be used for patients with resistance to fluconazole or who are not responding to topical treatment [82,83].

Issues arise when patients self-diagnose and then self-medicate and use products without proven efficacy against VVC [84]. Another issue that occurs is with over-the-counter drugs that pharmacist prescribes without medical consultation. Studies have revealed gaps in knowledge regarding the accurate identification of the cause of VVC; therefore, there is a need to update the guidelines to educate all medical staff [85].

### 3.6. Special Conditions

Several specific conditions require careful consideration when administering therapy [46]. For example, immunodeficient patients (e.g., those with poorly controlled diabetes), immunocompromised patients (HIV), and patients on immunosuppression therapy (corticosteroids) may not respond as well to short-term therapies; they should be put on treatment for prolonged periods [46] (Table 4). Due to the pathophysiological changes in HIV, diabetes mellitus and hormonal changes in pregnancy, these conditions are described in more detail, and most guidelines focus on them for this reason.

#### 3.6.1. Pregnancy

Vulvovaginal candidiasis increases during gestation due to immunologic alterations, higher estrogen levels, and increased vaginal glycogen production [91]. The most common causative agent is *C. albicans*, followed by *C. glabrata*, *C. tropicalis*, *C. krusei*, and *C. parapsilosis*; these non-albicans species generally cause milder symptoms in pregnancy than *C. albicans* [51]. As mentioned previously, *Candida* spp. colonize the vagina in around 20% of all women and over 30% of pregnant women [91]. Therefore, VVC infections are more prevalent in pregnant women, and most of the symptoms occur during the second and third trimesters of pregnancy [91,92]. Some research studies indicate that during pregnancy, symptomatic VVC is more likely; on the contrary, other studies found a higher prevalence of asymptomatic infections [91]. Increased estrogen levels enable *Candida* to transform from yeast into their invasive filamentous form, particularly by promoting the production of candidalysin, a toxin peptide of *C. albicans* with a cytotoxic effect on host cells, that promotes invasion and recruitment of leukocytes [51]. The pathogenic processes of *Candida* infections during pregnancy are also influenced by mannoproteins, which enable *Candida* to adhere to the vaginal epithelium, and by aspartate secretory proteinases that play a role in protein hydrolysis [51]. Risk factors also include multiparity and protracted use of antibiotics [51].

Recently, studies have focused on the potential impact of VVC on the fetus [51,91,92]. The studies suggested that vulvovaginal candidiasis may increase the risk of premature membrane rupture (which if prolonged may lead to chorioamnionitis), preterm labor, and congenital cutaneous candidiasis, contributing to host factors and intrauterine inflammation caused by the mother’s VVC [51,91]. These adverse perinatal outcomes may arise due to hormonal and immunological changes during gravidity [92]. Another contributor is a microbial invasion of the amniotic cavity, although, for congenital fetal candidiasis and candida amnionitis, transmission during childbirth and vaginal delivery are more plausible [51]. Hyperglycemia is a known contributing factor for *C. albicans* proliferation. Thus, it is no wonder that in prematurely born babies, systemic colonization happens three times more often when the glycemia levels are elevated [51]. There is some evidence in the medical literature that gestational diabetes mellitus might increase the risk of congenital *Candida* infections [51]. High-risk outcomes for the child, such as preterm birth or similar outcomes, were fortunately not demonstrated in the literature [92]. All this, however, points to the importance of an early and correct diagnosis that leads to sufficient therapy.

When opting for the optimal treatment approach, paying attention to the possibility of teratogenesis that various classes of medications can prompt is important. Hence, only topical azoles are recommended for a week [46,51]. It is imperative to apply antifungal therapy, emphasizing the last trimester and closer to birth, to avoid the possibility of transmission during delivery [51]. The evidence for this is the reduction rates of oral thrush and diaper rashes in newborns, from 10% to 2% in the first four weeks of life [51]. Naturally, symptomatic VVC requires treatment, most frequently 500 mg of clotrimazole applied intravaginally for around a week, as stated in the recommendations and guidelines [51]. Fluconazole, administered orally (doses of around 150 mg), has been linked to spontaneous abortions and congenital anomalies such as fetal cleft lip, transposition of the great vessels, and Fallot’s tetralogy [46,51]. Nevertheless, treatment of asymptomatic VVC during the first six months of pregnancy is not necessary [51].

#### 3.6.2. HIV Infections

Notwithstanding many outstanding therapeutic accomplishments of current treatments for HIV, VVC is still a common opportunistic infection in HIV seropositive patients, with much higher rates than among those without HIV with similar demographic backgrounds and risk behavior characteristics [46,93]. Symptomatic vulvovaginal candidiasis is also more common among women with HIV infection. It correlates well with the severity of immunodeficiency, with the most common cause of VVC in these patients being *C. albicans* [46,93]. Decreased levels of CD4+ T-cell counts have been associated with higher rates of VVC, and VVC has been linked to increased viral shedding [94].

According to the treatment guidelines, therapy for both uncomplicated and complicated VVC is akin to the treatment courses for patients without HIV [46]. Therefore, azoles remain the drug of choice for uncomplicated VVC [94]. In complicated VVC, chronic prophylaxis with fluconazole is adequate but not recommended for uncomplicated VVC [46]. Yet, it is still uncertain how the treatment of vulvovaginal candidiasis affects HIV acquisition and transmission [46]. As stated by the British Association’s HIV national guidelines for treatment of VVC, intravaginally administered imidazoles and oral azoles have success rates of 80% in acute VVC [95]. The recommended treatment for severe VVC is fluconazole 150 mg, and for recurrent VVC, an induction regimen is necessary for clinical remission, promptly followed by a maintenance regimen [95].

#### 3.6.3. Diabetes Mellitus (DM)

Diabetic women have higher rates of VVC and recurrent VVC compared to their nondiabetic peers, and studies also show that VVC occurs in type 1 diabetes mellitus more frequently than in type 2 diabetes mellitus [90,96,97]. This makes sense since hyperglycemia in the mucous membranes of the vagina and vulva is an excellent “breeding ground” for yeasts [98]. Hyperglycemic conditions impair neutrophil function, thus reducing phagocytosis and the clearance of *Candida* spp. [90,99]. Diabetes mellitus increases *Candida* virulence, as it leads to increased urinary secretion of glucose, which serves as a source of nutrition for *Candida*, thereby aiding its ability to colonize the vulvovaginal area [90,100]. Another contributor is *Candida*’s ability to change the vaginal pH, making the environment susceptible to yeast growth [101]. The most commonly identified pathogenic *Candida* species in this condition is also *C. albicans* [90,102]. Elevated hemoglobin A1C (HbA1c) levels were also found in individuals with diabetes with vulvovaginal candidiasis compared to diabetic women without VVC [96]. Sodium-glucose cotransporter 2 (SGLT2) inhibitors, which are used to treat DM, also increase urinary glucose excretion, which leads to a higher prevalence of VVC [90,103].

When it comes to the treatment of vulvovaginal candidiasis, diabetic patients should be treated for a more extended period (for about a week or two) since they are likely to have complicated VVC [90]. Asymptomatic *Candida* infections do not require therapy [90]. It has even been suggested that routine periodical screening for vulvovaginal candidiasis should be available to diabetic patients [96]. As discussed earlier, azoles interact with other drugs [104,105,106]. This, in turn, leads to an issue when treating women with diabetes since fluconazole and itraconazole can increase sulphonylureas levels, consequently causing hypoglycemia [107]. Hence, the best way to prevent VVC and its recurrence is to properly manage diabetes [98,108].

### 3.7. Prevention

Having outlined the clinical presentation, diagnostic approach, and therapeutic strategies for VVC, it is imperative to emphasize the primary and most effective measure—prevention. Preventing VVC, or at least lowering the odds of its occurrence, is possible by wearing cotton underwear, opting for breathable, non-restrictive clothing, and maintaining proper hygiene of the vulvovaginal area by keeping it clean and dry [109]. It is also important to avoid local irritants and scented products in the vaginal area and use emollients instead of soaps [107,110]. Douching should be avoided, as it removes the natural vaginal microbiota that protects the vagina from infections [110]. For quotidian personal hygiene, agents with a balanced pH and hypoallergenic properties are recommended [111]. Unnecessary use of antibiotics should also be avoided [107,110]. Emerging evidence even points to the possibility of using probiotics as a preventive strategy for VVC [112,113,114]. For example, *Lactiplantibacillus plantarum*, a dominant species in vaginal microbiota, has been shown to prevent pathogen adhesion and overgrowth when administered as vaginal capsules [115,116,117]. All this points out the high importance of proper patient education.

## 4. Possible Future Treatment Directions

Because of the ever-growing resistance to antifungal drugs, new medical approaches are much needed. Thus, numerous novel studies and preclinical trials have been conducted. New antifungal agents have recently been introduced to the market. Ibrexafungerp, a triterpenoid antifungal that was the first (FDA) approved oral non-azole agent for VVC, and is currently only available for this purpose in the USA [118]. Inhibiting glucan synthase disrupts the formation of 1.3-β-D-glucan (a primary part of the fungal cell wall), which leads to cell lysis [119]. Ibrexafungerp is effective in treating severe and recurrent VVC, but the side effects include nausea, vomiting, diarrhea, dizziness, and abdominal pain [118]. The recently approved oteseconazole is effective in both acute and recurrent VVC [120]. Since the mechanism of action works based on a highly selective inhibition of the fungal CYP51 (over human CYPs), it has lower toxicity [121].

Many attempts have been made to develop various types of antifungal vaccines for *C. albicans*. A live attenuated vaccine was developed based on the genetically altered *C. albicans* tet-NRG1 strain, which is regulated by the amount of doxycycline, thereby changing its morphology and virulence [122]. Since recombinant vaccines are easier to administer and contain no infectious organisms, they are significantly safer than their counterparts [123]. Studies on rats that received recombinant aspartyl proteases, Sap2, intravaginally showed a reduction in *Candida* vaginal infection [124,125]. A similar study with Sap2, encompassed by influenza virosomes (PEV-7), found elevated levels of antibodies in vaginal fluid in mice and rats following intravaginal, intramuscular, and combined administrations. Thus, the authors suggested clinical trials of the vaccination for RVVC [126]. Synthetic glycopeptide conjugate vaccines are effective against *C. albicans;* however, they are expensive and challenging to synthesize [127,128]. Vaccination with Als3p-N protein of *C. albicans*, fused with alum adjuvant has shown to be effective in preventing VVC [129]. However, inactivated vaccines, containing heat-killed *C. albicans* and genetically altered toxins as adjuvants, have not been able to raise immunity in animal models when administered intravaginally [130].

Probiotics are considered a natural approach to prevent and treat vaginal infections, as some probiotics contain *Lactobacillus* species that have an antagonistic effect on *Candida*-induced vulvovaginitis [80]. It is no wonder, then, that they have achieved remarkable results in their fight against VVC [48,115,131,132]. In vitro models have shown a significant reduction in fungal colonization after treatment with probiotics, such as *Lactobacillus rhamnosus* GR-1, *Lactobacillus reuteri* RC-14, or their combination [133]. A study comparing the effects of *Lactobacillus rhamnosus* GR-1 and *Lactobacillus fermentum* RC-14 against a placebo found no side effects, a return to normal microflora from asymptomatic bacterial vaginosis, and a reduction in yeast [134]. Probiotics appear to be a contentious substitute for azoles. A 2021 study compared topical clotrimazole treatment versus oral clotrimazole that contained live strains of *Lactobacillus acidophilus* GLA-14. The group treated with the probiotic had a higher success rate, fewer adverse effects, and lower relapse rates [135]. Another study found that combining fluconazole with probiotics increases the azole’s efficiency by decreasing the overall amount of yeast and mitigating symptoms in patients diagnosed with VVC [136]. Nonetheless, the Centers for Disease Control and Prevention’s guidelines unequivocally state that there is insufficient evidence to support the use of probiotics in the treatment of VVC [137].

## 5. Conclusions

Since *C. albicans* is a natural human commensal, it is no wonder that the number of infections it causes continues to rise year after year. This increase may be attributed to a combination of factors, including a more stressful lifestyle, widespread use of antibiotics, increasing antifungal resistance, and a growing number of individuals with underlying risk conditions. An increasing number of women are affected by vulvovaginal candidiasis annually, with significant implications for their quality of life. Vulnerable groups (like older individuals or pregnant women, people with diabetes, and people living with HIV) are especially susceptible and, thus, should remain vigilant. Proactive measures to strengthen their immune defenses, as well as maintaining proper hygiene and microbiome balance, are key to preventing recurrence and complications. Due to the ever-growing resistance to current antifungal therapies, there is a pressing need to explore new therapeutic strategies. Furthermore, additional studies are needed to evaluate the safety, efficacy, and mechanisms of alternative treatments, particularly natural remedies and plant-based compounds that have shown promising antifungal potential in preliminary research. Reducing the burden of VVC definitely requires a comprehensive public health approach that prioritizes awareness, timely diagnosis, equitable access to effective treatment, as well as continued research endeavors.

## Figures and Tables

**Table 1:** Common *Candida* species as etiological agents of vulvovaginal candidiasis (VVC) [6].

*C. albicans*	Non–Albicans
	*Nakaseomyces glebaratus* (*C. glabrata*)
	*Pichia kudriavzevii* (*C. krusei*)
	*C. parapsilosis*
	*C. tropicalis*

**Table 2:** Vulvovaginal candidiasis classification and symptoms.

Classification:	Uncomplicated	Complicated
	sporadic or infrequent VVC mild-to-moderate VVC	recurrent VVC severe VVC non-albicans VVC
Symptoms: vaginal soreness, genital pruritus, burning sensation, irritation, dyspareunia, dysuria, an abnormal vaginal discharge, erythema of the vulva and vagina, edema	Mild to moderate symptoms	Severe symptoms (extensive vulvar erythema, prominent edema, excoriation, and fissure formations)
Frequency:	<4 times a year	>4 times a year

**Table 3:** Medical treatment of VVC [46].

Name	Type of Medicine	How to Use	Duration of Treatment
Clotrimazole 5 g	1% cream	intravaginally	daily for 7–14 days
Clotrimazole 5 g	2% cream	intravaginally	daily for 3 days
Miconazole 5 g	2% cream	intravaginally	daily for 7 days
Miconazole 5 g	4% cream	intravaginally	daily for 3 days
Miconazole 100 mg	vaginal suppository	intravaginally	one suppository daily for 7 days
Miconazole 200 mg	vaginal suppository	Intravaginally	one suppository for 3 days
Miconazole 1200 mg	vaginal suppository	intravaginally	one suppository for 1 day
Tioconazole 5 g	6.5% ointment	intravaginally	a single application
Butoconazole 5 g	2% cream	intravaginally	a single application
Terconazole 5 g	0.4% cream	intravaginally	daily for 7 days
Terconazole 5 g	0.8% cream	intravaginally	daily for 3 days
Terconazole 80 mg	vaginal suppository	intravaginally	one suppository daily for 3 days
Fluconazole 150 mg	capsule	orally	a single dose
Oteseconazole	capsules	orally	day 1: 600 mg (single dose), day 2: 450 mg (single dose), day 14: 150 mg once a week (every 7 days) for 11 weeks
Ibrexafungerp	300 mg tablets	orally	two tablets of 150 mg twice a day for 1 day

**Table 4:** The condition needed to be taken into consideration when it comes to vulvovaginal candidiasis.

Condition	Modes of Transmission	Clinical Outcomes	Risk Assessments	Diagnostic Techniques	Interpretation Challenges	Ref.
Pregnancy	due to immunologic alterations, higher estrogen levels, increased vaginal glycogen production	premature membrane rupture, preterm labor, chorioamnionitis, congenital cutaneous candidiasis	30% of pregnant women	Wet mount vaginal swab of the vaginal contents to complement the clinical examination	Controversy regarding the safety of oral antifungals during pregnancy	[86]
HIV	Opportunistic infection correlates with the severity of immunodeficiency	decreased levels of CD4+ T-cells	Higher rates than among those without HIV Prevalence is correlated with the immunological status of the host	microscopy, yeast culture, clinical examination	higher incidence and greater persistence, but not greater severity	[59,87,88,89]
Diabetes mellitus	decreases in leukocyte chemotaxis and phagocytosis, impairment in vascular reaction	may also develop as an adverse effect of treatment with sodium–glucose cotransporter 2 (SGLT2) inhibitors	Higher prevalence rates than among those without DM	Based on clinical evidence, vaginal or swab test	highly prevalent non-infectious vulvovaginal conditions: skin disorders (e.g., acanthosis nigricans, skin tags, and vitiligo), pelvic organ prolapse, and pelvic pain	[90]
